# Quantitative selection of focal birds and mammals in higher‐tier risk assessment: An application to rice cultivations

**DOI:** 10.1002/ieam.4535

**Published:** 2021-11-03

**Authors:** Valerio Orioli, Alessandra Caffi, Flavio Marchetto, Olivia Dondina, Luciano Bani

**Affiliations:** ^1^ Department of Earth and Environmental Sciences University of Milano‐Bicocca Milan Italy; ^2^ ICPS, International Centre for Pesticides and Health Risk Prevention, ASST Fatebenefratelli‐Sacco Milan Italy; ^3^ Syngenta Italia S.p.A. Milan Italy; ^4^ European Chemical Agency Helsinki Finland; ^5^ World Biodiversity Association Onlus c/o NAT LAB Forte Inglese Portoferraio, Livorno Italy

**Keywords:** Ecological traits, Expert‐based model, Generalized additive model, Kriging interpolation model, Pesticide registration

## Abstract

European Pesticide Registration requires a risk assessment (RA) for nontarget organisms according to EU Regulation. European Authorities have developed Guidance Documents (GDs) for RA considering exposure scenarios for the required organisms typical for terrestrial crops. The “Birds and Mammals EFSA GD” allows using multiple sources of information to extract information on species frequency needed in identifying focal species for higher‐tier RA. We developed an analytical framework to calculate species frequency according to availability of species and habitat quantitative data. Since the exposure scenarios reported in the EFSA GD are inconsistent for rice, we tested the method on birds and mammals in a portion of the largest rice‐cultivated area of Europe, the Italian Po floodplain. We derived three lists of focal species: (a) an expert‐based list based on land‐use data only, which can be useful for a preliminary exploration of potential candidate species; (b) a list derived from the interpolation of species data only, which reflects actual species frequency in rice fields; and (c) a list obtained by a species distribution model based on species monitoring and land‐use data, which account for species selectivity for rice crops and are transferable to other contexts. Focal species were identified for crop‐specific diet‐foraging guilds, to build specific exposure scenarios to assess the risk from pesticides application in rice fields. The partial differences between our lists and those previously proposed highlight the need for identifying national lists, which can vary according to study area, biogeographic region and exposure scenarios. The application of the proposed method in European rice‐producing countries should lead to crop‐specific lists, which could then be integrated to obtain a flexible European list applicable to higher‐tier RA. *Integr Environ Assess Manag* 2022;18:1020–1034. © 2021 The Authors. *Integrated Environmental Assessment and Management* published by Wiley Periodicals LLC on behalf of Society of Environmental Toxicology & Chemistry (SETAC).

## Key Points

- We provided an analytical framework for improving selection of focal species for higher‐tier risk assessment of pesticides in rice‐cultivations and any other crop.
- Expert‐based models should be used for preliminary exploration of focal species, spatial interpolation of species occurrences should be used for local assessment, while species distribution modelling provides information transferable to other contexts.
- We identified the Black‐winged Stilt and the Northern Lapwing as focal species for water insectivores, the Little Egret for water carnivores, the Moorhen for water herbivorous birds and the Barn swallow and the Northern House‐martin for aerial insectivorous birds.
- We identified the Eurasian harvest mouse as focal species for water insectivorous and herbivorous mammals, the Eurasian water shrew for water carnivorous and piscivorous mammals, and the Lesser horseshoe bat for aerial insectivorous mammals.

## INTRODUCTION

The European Regulation 1107/2009 EC describes the rules for Pesticide Registration to develop a risk assessment (RA) for nontarget organisms. European Authorities drafted Guidance Documents (GDs) to harmonize procedures and perform a proper RA by comparing the substance toxicity with organisms' exposure. Substance toxicity is derived from toxicological studies, whereas the exposure is estimated using models and assumptions (Dietzen et al., 2014). A key concept of RA is the principle of the “tiered approach,” by which each step represents a worst case compared to the following one. RA is characterized by a stepwise evaluation represented at the beginning (lower tiers) by low realism and complexity. These steps represent a highly protective assessment of the risk. If the assessment at the lower tiers results in a high risk, it is possible to consider more complex scenarios close to reality in the higher‐tier RA. In this step, RA is performed on species actually occurring in the target crop during the period of pesticides application (Schäfer et al., 2019).

In the latest version of the GD for RA on birds and mammals, developed by the European Food Safety Authority (EFSA, 2009; hereafter, we refer to this document as “EFSA GD”), the dietary exposure to pesticides is the most relevant route of exposure. Based on this consideration, birds and mammals are grouped into “feeding guilds”: species diets are combined with food items potentially contaminated from pesticides and collected in treated fields. The lower‐tier feeding guilds are represented by “indicator species” (screening step) or “generic focal species” (first tier). These are “surrogate species” that belong to the same feeding guild for which the ratio between Food Intake Rate and body weight (FIR/bw) is the highest among all European species potentially occurring in a specific crop at a particular time. In the higher‐tier assessment, exposure can be refined by identifying “focal species” for crop‐specific feeding guilds among those species actually occurring in the target crop within the area of assessment (Dietzen et al., 2014).

The exposure scenarios reported in the EFSA GD have been developed for terrestrial crops, while the assessment for rice cultivations is not included in the GD and is left to a specific Guidance, which is currently in preparation. Indeed, rice cultivation is performed in semiaquatic conditions: a water layer is maintained in rice paddy to buffer the temperature differences during the growing period (Confalonieri et al., 2005), variably from presowing (late March to early April) to preharvest (late August to early September) (Ferrero & Tinarelli, 2008). This condition allows comparison of rice fields to shallow temporary water bodies (Fasola & Ruiz, 1996; Lawler, 2001; Natuhara, 2013). Exposure scenarios described in the EFSA GD in use are therefore inconsistent for rice and suggested that focal species for higher‐tier RA are unlikely to be representative of and protective for vertebrate communities exposed in rice fields in Europe.

Bird and mammal communities related to paddy fields are consistently different from those related to other crops due to the agricultural practice of flooding paddies and the different supply of food items (Longoni, 2010; Tourenq et al., 2001). Avifauna in rice‐cultivated areas is characterized by a deep seasonal variability in species composition. In early spring, flooded rice fields host a great variety of species (waders, ducks, gulls, herons and some passerines), which use this environment as a stop‐over area for refreshment during spring migration toward northern breeding sites (Elphick, 2010). In late spring and summertime, paddy fields usually host water birds such as ducks (Pernollet et al., 2015), the herbivorous Common Moorhen (*Gallinula chloropus*) and the insectivorous Northern Lapwing (*Vanellus vanellus*) or herons that feed on water vertebrates (amphibians and fish). These species use paddies for feeding and for nesting in their proximity (Fasola & Ruiz, 1996; Lourenço & Piersma, 2009). Moreover, many passerines can feed in rice paddies from the water surface, from emerging ground when water is shallow and from the perimetral ground layer (e.g., Motacillidae) or from the aerial layer over water (e.g., Hirundinidae) (Ibáñez et al., 2010). At the end of summer, when water is usually removed, rice ripens and the threshing phase begins, paddies represent the proper environment for several granivorous species. They are attracted by rice grains that remain on the field surface after the mechanized harvest (e.g., Wood Pigeons and small passerines like chaffinches).

Flooded rice fields represent a less suitable environment for mammals compared to birds and therefore a lower number of species frequent this habitat (e.g., rodents and insectivores; Bambaradeniya & Amerasinghe, 2004; Bambaradeniya et al., 2004). However, less information on the distribution and abundance of these organisms is available due to technical difficulties in conducting field studies in flooded fields and the need to use specific survey techniques according to mammalian taxa (Vallon et al., 2018).

Considering the above, an analysis of bird and mammal communities is necessary to identify focal species able to depict appropriate exposure scenarios for higher‐tier pesticide RA in rice fields.

An attempt to propose focal species for RA in European rice fields has been published by Vallon et al. (2018) by reviewing existing literature data and consulting experts. However, this review presents some limitations: the analysis is semiquantitative, based on the results of previous publications, which were mainly focused on nonpasserine orders of birds; no data on the major rice‐producer country in EU (Italy, ~53%, https://agridata.ec.europa.eu/extensions/DashboardRice/RiceProduction [accessed 19 July 2021]) are reported; and the selection of focal species has been conducted according to the EFSA GD diet guilds considering rice as belonging to the “cereal” crop group. As stated by the authors, to solve these issues, quantitative data on the whole rice fields' community are needed and guilds should be selected according to the specificity of the diet of paddy‐dwelling species.

The analysis of species frequency for the identification of representative species is usually conducted by field studies designed with this precise purpose, as suggested by EFSA GD (Appendix M). Alternatively, the analysis can be performed using literature and/or survey and census data as also stated in the EFSA GD: “Other data that may be used to determine focal species may include survey or census information.” Indeed, several authors adopted different sources of information from literature (Vallon et al., 2018) or survey and census data (Crocker & Irving, 1999; Gandolfi & Reichlin, 2012; Sabo et al., 2010) to identify focal species for RA. However, these data require alternative analytical methods with respect to those suggested by EFSA GD to calculate species frequency in the target area. In particular, species distribution models (SDMs) were found to be effective in predicting species occurrence from quantitative census data (Massimino et al., 2008). The combination of data type and analytical approach provides different pieces of information in the process of selecting focal species: (a) raw data from properly designed studies or representative surveys within a target area indicate the actual composition of the local community; (b) SDMs developed on such data or even less representative data sets (e.g., monitoring data at regional scale) indicate the potential composition of the community, while accounting for species–habitat relationships. Species distribution model results can be exploited to produce either local‐ or regional‐scale predictions and selection of focal species for areas with consistent communities; (c) when quantitative data are not available, semiquantitative models, where species–habitat relationships are deduced from experts' opinions or literature (e.g., Vallon et al., 2018), can be constructed.

In the present study, we developed a general analytical framework to identify sets of focal species for higher‐tier RA that can be potentially applied to any crop type and study area, when data other than those suggested in Appendix M of EFSA GD are available. The method handles multiple sources of information, from quantitative field data to a semiquantitative expert‐based approach. We applied the method to propose national sets of focal species for higher‐tier RA in rice cultivations in Italy according to available data. We analyzed communities of breeding birds and mammals using predictive models applied to survey, census or literature data to establish the prevalence of species occurring in rice fields during the main period of pesticide exposure (Comoretto et al., 2007; Karpouzas et al., 2006). On the basis of the available information, we used different models to obtain maps of actual (survey data only) or potential (survey and habitat data) distribution or relative suitability (habitat data only) for each species. We overlapped these maps to a land‐use surface to filter species that effectively use rice fields. We later allocated species to different feeding guilds by considering differences in diet composition and foraging stratum. Finally, we identified the lists of focal species for higher‐tier RA in rice cultivations on the basis of the food items available in rice fields and body weight.

## METHODS

### Study area

**FIGURE 1:**
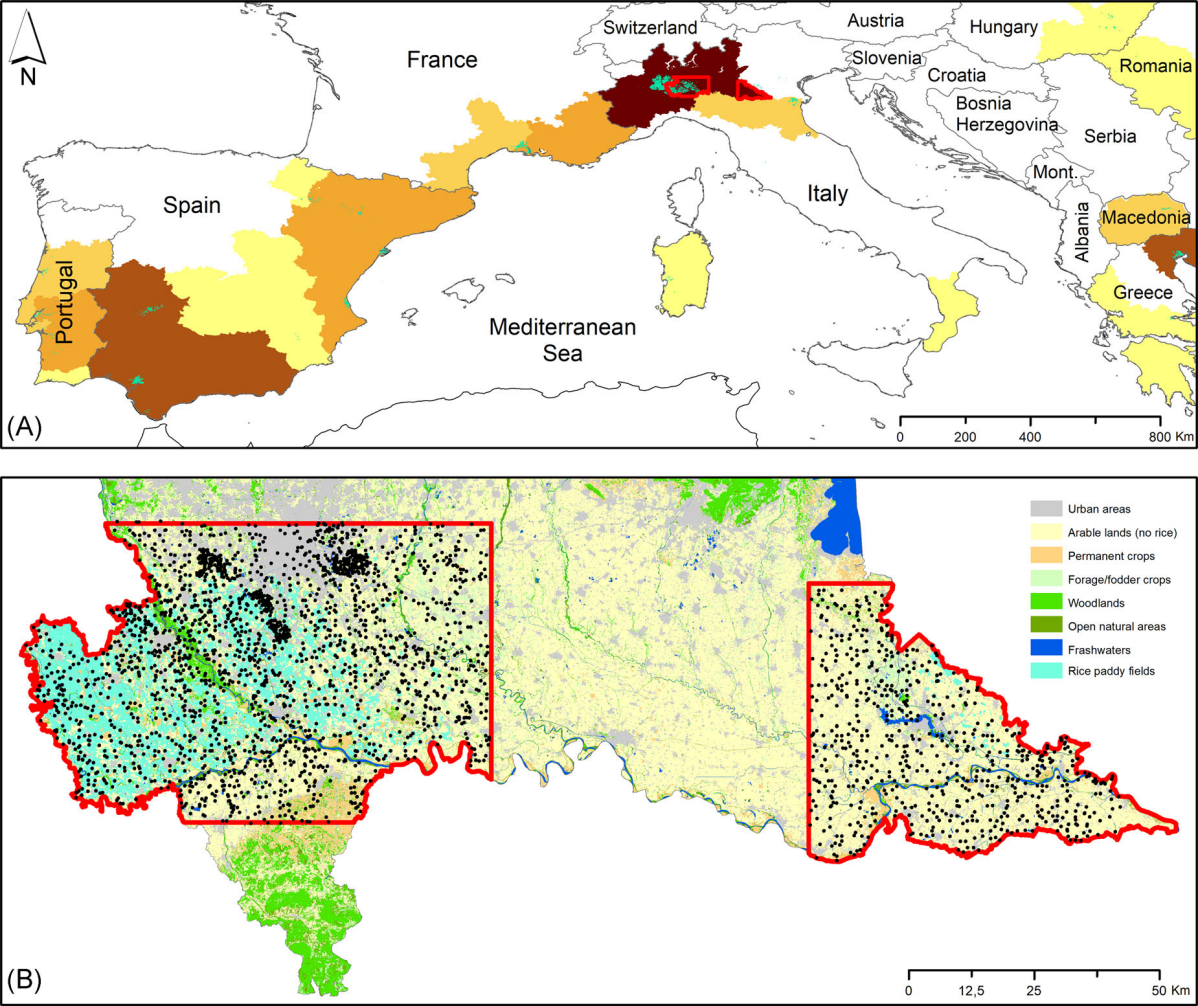
Study area and bird data. Location of the study area within (A) the Mediterranean Region and (B) the Lombardy NUT2 Region in Northern Italy. Rice cultivations are shown in light blue (adapted from Corine Land Cover, 2018), and study area borders are in red in both boxes. In (A) NUT2 European Regions are filled with colors proportional to the extension of rice cultivations (based on data coming from Laborte et al., 2017; class 1 (lighter) < 3312 ha, class 2 3312‐11130 ha, class 3 11130‐20695 ha, class 4 20695‐38311 ha, class 5 (darker) > 38311 ha). In (B), background colors indicate land‐use classes (DUSAF 5.0; ERSAF, 2015) and dark dots show the locations of bird counts performed in 2000–2016

The study was conducted in the central part of the Po river plain within the Lombardy NUT2 European Region, one of the most human‐modified areas of EU (EUROSTAT, 2020; Figure 1A). Forest cover amounts to 24.6% of the region surface (DUSAF 5.0; ERSAF, 2015), wherein broad‐leaved forests are highly fragmented (95% of fragments smaller than 10 ha; Dondina et al., 2017) and intensively managed (94% managed as coppice; Dondina et al., 2015). Agricultural areas cover 42% of the region surface (DUSAF 5.0; ERSAF, 2015). Forage and fodder crops, maize and other cereal grain crops are the most widespread crops in the Region (ISTAT, 2018), which also hosts more than 40% of the widest area assigned to rice production in Europe (i.e., the Italian Po River Plain; EUROSTAT, 2020; Laborte et al., 2017; Figure 1A). The study area embeds two disjunct subareas 4498 and 2094 km^2^ wide, located in the western and eastern parts of Lombardy (Figure 1B). Both areas are mainly composed of agro‐ecosystems (73.8% of the western and 82.4% of the eastern area), but with a different allocation of crop types. Rice paddies occupy 26.8% of the western subarea, followed by maize 23.9%, forage and fodder (14.8%), cereals (7.3%), and other crops (e.g., soybeans and rapeseed; 7.2%). The eastern subarea is composed of a lesser cover of rice fields (0.6%), while other agro‐ecosystems are allocated to maize (25.4%), forage and fodder (24.8%), cereals (16.1%), and other crops (10.0%). We did not include the interspersed territory between the two subareas, where rice is not cultivated, to reduce the effect of zero‐inflated counts (true zeros) of paddy‐dwelling species on the results (Denes et al., 2015).

### Data collection

#### Bird and mammal data

Bird data were collected in the whole Lombardy Region between 2000 and 2016, except in 2004, 2005, and 2007, when data were collected only from a portion of the Region and were thus inadequate for the aims of the present study (see Bani et al., 2009 for details). We drew sampling units according to a stratified two‐stage design, whereby the Lombardy region was subdivided into seven macro‐areas with a homogeneous land‐use composition, within which first‐stage 10 km^2^ sampling units (~20% of the region) were randomly chosen. Survey points were located in 1 km^2^ second‐stage sampling units (~15, according to accessibility) randomly extracted within first‐stage units. The sampling design was renewed yearly to increase the whole‐period sampled area, so that single sampling points were not surveyed for 2 consecutive years (but they could have been replicated after some years due to chance). Surveys were performed from 10 May to 20 June to include the breeding season of all species in the area, which corresponds to the main flooding period of rice paddies. In each sampling point, bird presence was collected by a 10‐min point count, recording all seen and heard bird species (Bani et al., 2009). Overall, we collected data on 143 bird species, belonging to 17 orders and 50 families (Table S1).

Quantitative data of mammals were not available for the study area, neither were updated and representative indirect data, such as those from atlases or citizen science projects. In particular, we were unable to use data contained in the atlas of mammals of Lombardy (Prigioni et al., 2001), as species distributions were aggregated at 10 km^2^, which was too coarse a resolution for the purposes of our study. Therefore, we used an expert‐based approach to identify focal species for mammals (see Data analysis section).

#### Land‐use data

Wildlife distribution is strongly affected by availability of suitable habitats where animals can breed and find shelter or food resources (Grinnellian niche concept; Hirzel & Le Lay, 2008). In our modeling framework, we considered land‐use composition as a surrogate of habitat availability for target species. Bird distribution models were developed using the land‐use fractional cover in a 250 m radius buffer around each sampling point, collected in the field by visual inspection. The radius is representative of the average distance within which most of the bird species can be detected (detecting radius; Bani et al., 2009).

Species distribution models for birds and expert‐based models (EBM) for both birds and mammals were used to spatially predict species distribution by means of digital land‐use data extracted from the most recent land‐use map of Lombardy within the study period (DUSAF 5.0; ERSAF, 2015). We assumed that land‐use changes that occurred in 1999–2015 (Table S2) did not affect species ecology and selectivity for rice‐cultivated areas. For each land‐use class (Table S3), we produced a raster map by assigning to each pixel (sized 20 × 20 m) the fractional cover of the class within a 250 m buffer around the pixel to match the sampling area defined by the detecting radius. We also produced two maps expressing the latitudinal and longitudinal positions by assigning to each pixel the North and East coordinates of the pixel, respectively, to account for possible spatial autocorrelation in species occurrence data (see below).

### Data analysis

**FIGURE 2:**
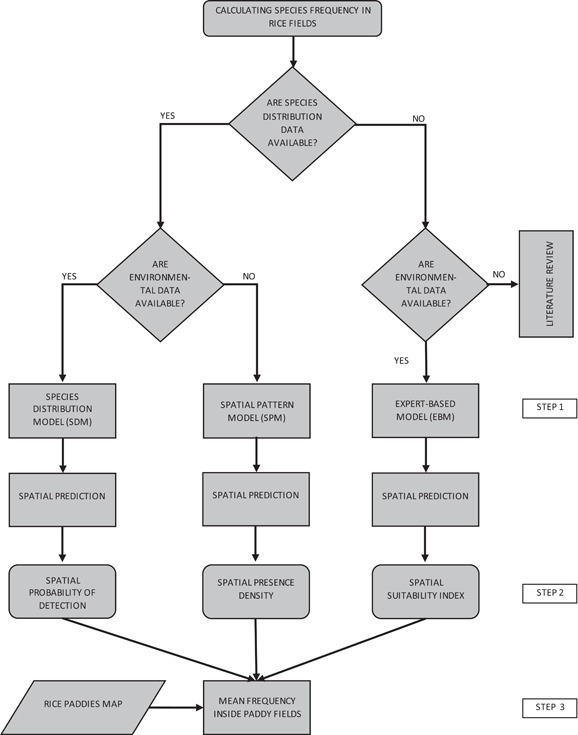
Conceptual diagram of the calculation of species frequency in rice fields. Step 1: choice of the proper modeling approach according to the availability of habitat data, species detection data or none of these. Step 2: spatial prediction of species frequency, according to the chosen modeling approach. Step 3: calculation of the mean species frequency in rice fields by overlapping the spatial location of paddies

The higher tier of the RA procedure for birds and mammals requires the evaluation of species frequency in target cultivations (EFSA GD, Appendix M). To select bird focal species, we compared three approaches that differ for the type of data they demand (see Graham & Hijmans, 2006): an expert‐based approach requiring habitat data only (EBM), an interpolation method based on species detection data (spatial pattern model, SPM) and a SDM requiring both species detection data and habitat data. In our case, we used land‐use classes of the available digital map (see above) as habitat data. To select mammal focal species, we applied the EBM to paddy‐dwelling mammals, for which exhaustive distribution data are lacking in the study area (Figure 2—Step 1). Each model was used to produce a spatial prediction on a raster surface (pixel size 20 × 20 m) that provides different information according to the analyzed data and modeling approach: EBM prediction indicated a normalized suitability index. SPM prediction was a density surface representing the mean number of presences per pixel. Species distribution model prediction was a map indicating the probability of detecting the species in each pixel (Figure 2—Step 2). All indexes ranged between 0 and 1. For each modeling approach, we finally expressed species frequency inside rice paddies as the mean value of those pixels of prediction maps falling inside rice paddies (DUSAF 5.0; ERSAF, 2015; Figure 2—Step 3). Apart from input constraints and statistical assumptions, the three modeling approaches provide different implications for the RA procedure. Expert‐based model outputs lead to the selection of focal species valid in general on the basis of known species ecology, which cannot eventually be representative of the local community of rice‐dwelling species. Conversely, focal species identified by the SPM approach reflect the local community composition but are not generalizable to other contexts. Species distribution model outputs provide focal species that are both valuable for the local context and are transferable to similar environmental contexts, such as large regions with extensive rice cultivations.

To compare model performance, we calculated Pearson's correlation coefficient between the output raster maps of the three models for each bird species and the mean correlation across species for the three pairwise comparisons.

#### Model 1: EBM

When species distribution data are lacking for a specific area, expert‐based range assessment is a possible yet criticized solution (Fourcade et al., 2013; Martin et al., 2012). Expert knowledge can be involved in multiple steps of the species range assessment, from providing of species occurrence data to encoding distribution parameters (Fourcade, 2016; Martin et al., 2012). We developed mathematical equations describing habitat suitability as a function of a weighted combination of independent variables, whose weights were encoded according to knowledge about the ecology and the biology of the target species. We formulated three equations that relate the amount of different land‐use classes to a relative index of species presence. The equations describe three possible species–habitat relationships according to species selectivity for a given pair of habitat classes.

The first equation refers to an “internal” relationship (Equation 1), which occurs when habitat suitability linearly grows with increasing amount of a main habitat class (i.e., the habitat including essential resources, such as food, water, and shelter), reaching its maximum when habitat amount is 100%, while a secondary habitat class (i.e., every habitat bordering the main habitat) can determine the average suitability when the main class amount is close to zero (Figure S1a).

(1) $$S=C_{m}*\beta_{m}+C_{s}*\beta_{s}*C_{m}^{0,1},$$

where *S* is the suitability index, *C*_m_ is the amount of the main habitat class and $\beta_{m}$ is the coefficient of the main habitat class, while *C*_s_ and $\beta_{s}$ are the amount and the coefficient of the secondary habitat class, respectively.

The second and third equations describe “edge” relationships, which occur when a species prefers areas at the edge between two habitat classes (i.e., ecotone species), reaching its maximum at intermediate values of the two habitat classes. The equation can assume two forms: “internal edge” if maximum suitability is reached toward a greater amount of the main habitat class (Equation 2a, Figure S1b) and “external edge” if maximum suitability is reached for a greater amount of the secondary habitat class (Equation 2b, Figure S1c).

(2a) $$S=((1-C_{m})\times C_{m}^{\mathrm{Pow}}\times \beta_{\mathrm{int}}\times \beta_{m})\times \text{Pow}\;+\;(C_{s}\times \beta_{s}\times C_{m}^{0,1}),$$

(2b) $$S=((1-C_{m})\times C_{m}^{\mathrm{Pow}}\times \beta_{\mathrm{int}}\times \beta_{m}+C_{m}\times \beta_{m})/\mathrm{Pow}\;+(C_{s}\times \beta_{s}\times C_{m}^{0,1}),$$

where symbols are the same used in Equation (1), except for Pow, a power coefficient that describes the shape of the relationships, and β_int_, which expresses the strength of the interaction between the two habitat classes. The function parameters (all β and Pow) were set to express null (0), light (0.25), medium (0.5), high (0.75), or strong (1), positive or negative, effects of habitat classes on relative suitability. The effect of other covariates, that is, altitude, slope gradient, and aspect and river density, were included as a linear combination of the covariate amount or level (for categorical covariates) and an expert‐based coefficient. For each species, we encoded a complete equation by summing as many single equations as the number of pairwise habitat relationships for that species. For some species, a combination of internal and edge equations was used. The appropriate equations and its parametrization were chosen according to authors' expertise.

To map species' range in the study area, a pixel‐wise relative habitat suitability was calculated applying the species‐specific complete equations on the amount of different habitat classes (*C*_m_ and *C*_s_) within a 250 m buffer around each pixel, and the pixel value of altitude, slope, aspect and the presence of rivers. To standardize the suitability index and to compare its output to those obtained by the other two modeling approaches, its range was constrained between 0 (unsuitable) and 1 (highest suitability) by dividing the difference between a pixel value and the minimum pixel value by the difference between the maximum and the minimum pixel value.

#### Model 2: SPM

Kriging is a geostatistical method used to predict species spatial pattern (i.e., range and probability of occurrence) when observational data are available and environmental data are not available (Yalcin & Leroux, 2017). Spatial predictions are produced by interpolating point data values with a statistical model that represents spatial autocorrelation of data through a semivariogram. The latter is a mathematical function that describes the variability in the scores of the dependent variable within sequential distance classes. When the dependent variable has a binomial distribution (e.g., detection data including presences and absences), the prediction of a kriging interpolation is comparable to a detection probability. The general equation of kriging interpolation is as follows:

$$\hat{Z}\left(S_{0},)\right)=\sum_{i=1}^{N}\lambda_{i}Z(S_{i}),$$

where *S*_0_ is the prediction location, *Z(s*_*i*_ *)* is the measured value at the *i*th location, *λ*_*i*_ is a weight for the measured value at the *i*th location that depends on the spatial pattern estimated by the semivariogram, and *N* is the number of measured neighbor values.

To produce bird distribution maps, we used the ordinary kriging method (constant mean occurrence) with a spherical semivariogram model (spatial autocorrelation decreases until a certain distance, beyond which no autocorrelation is observed), and we predicted occurrence at unknown localities by using 30 neighbor points in a maximum radius of 5 km (variable search radius). Geostatistical analyses were performed using the *Kriging* tool in the *3D Analyst* toolbox of ArcGis 10.5.0.6491 (ESRI Inc., 2016).

#### Model 3: SDM

To quantify the relationships between species occurrence and land‐use cover, we used a generalized additive model—a regression technique that allows modeling of nonlinear effects of covariates on a non‐normal distributed response variable through an appropriate link function (Wood, 2017). We used the logarithm as the link function, which is more appropriate to model Binomial‐distributed random variables, such as the detection (1: present, 0: absent) of a species. We modeled nonlinear effects of covariates by including smooth spline functions of land‐use fractional covers (Table S3), with the upper limit of degrees of freedom (*k*) set at 4. We included a smooth function of year with a fixed *k* equal to 14 (the duration of the study in years) to model the possible interannual dynamic of species distribution. We also added a full tensor product smooth of longitude and latitude (both with *k* = 10) and year (*k* = 14) to account for the spatial and temporal autocorrelation of sampling points (Harrison et al., 2014; Wood, 2017). The complete models were reduced to more parsimonious models using a two‐step covariate selection procedure. We first removed all covariates with a value of estimated degrees of freedom lower than 0.1 and, second, we iteratively removed the least significant variable (highest *p*‐value) until all covariates had a *p*‐value lower than the critical value of 0.05 (backward stepwise selection). Analyses were performed in R environment (v4.0.3; R Core Team, 2020) using the *mgcv* package (Wood, 2019).

Species' range was mapped as the pixel‐wise probability of detection (range: 0–1) calculated by applying the final reduced model to the correspondent pixel value of the maps of geographical covariates and land‐use fractional cover in a 250 m buffer.

### Identification of focal species

**FIGURE 3:**
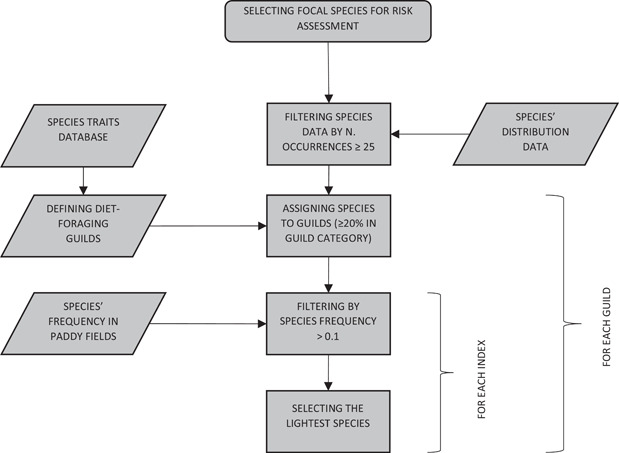
Conceptual diagram of the identification of focal species for risk assessment in rice cultivations. When quantitative data were available, species occurring in less than 25 points in rice fields were removed to avoid inference bias of rare species. Diet‐foraging guilds were defined using a quantitative species traits database (adapted from Wilman et al., 2014). Species were assigned to guilds when they used at least 20% of the corresponding diet and foraging resource. Species with a frequency in rice fields lower than 0.1 were removed. The species with the lowest body mass was selected as the focal species. Focal species selection was repeated separately for each guild and each frequency index

To identify focal species, we followed a tiered selection algorithm (Figure 3). When quantitative data were available (i.e., birds), we created a starting list composed of all species recorded at least once in our study area. We filtered the list by removing rare species detected in less than 25 points performed in rice fields (points with more than 5% of rice cover in a 250 m buffer), as modeling those species can be tricky due to the presence of a large amount of zeros (Denes et al., 2015; Wenger & Freeman, 2008). We used the same filtered list to perform all three modeling approaches. When no quantitative data were available (i.e., mammals), we considered all species potentially occurring in the study area (Vigorita & Cucè, 2008).

We defined rice‐specific diet‐foraging guilds (DFGs) on the basis of the EltonTraits 1.0 database compiled by Wilman et al. (2014). First, we identified all possible DFGs included in the database, and successively, we chose only those relevant for rice cultivations. The database includes quantitative data on species traits of world birds and mammals, which are encoded as relative importance (percentage of use with a 10% step approximation), on the basis of a standardized interpretation of the literature. Specifically, the bird database provides for 10 categories of diet (invertebrates, endothermic vertebrates, ectothermic vertebrates, fish, unknown vertebrates, scavenges, fruits, nectar, seeds, plants) and seven categories of foraging stratus (below the water surfaces, around the water surface, ground, understorey, mid‐high, canopy, aerial). The mammal database includes the same diet categories of the bird database, while foraging stratus is defined by a categorical factor with five levels (marine, ground [including water], scansorial, arboreal, aerial). Both databases also include the species body mass, calculated as the geometric mean of the average values provided for both sexes for birds and the average adult body weight for mammals. We grouped the diet categories into seven classes by merging the vertebrates' categories into “Vertebrates” and fruit and nectar into “Fruits.” We grouped the foraging stratus categories into four classes by merging the water classes into “Water” and understorey, mid‐high and canopy into “Vegetation.” We merged all vegetation strata into one category, because they represent secondary habitats in rice paddies. We modified the definition of the aerial foraging stratus. We considered as aerial feeders those species feeding at any altitude above ground and/or water, while the EltonTraits database assigned species to the understorey, mid‐high, canopy, and aerial category according to the height from ground where main foraging occurs (<2, ≥2 m, into the canopy and well above the canopy, respectively). We assigned scansorial and arboreal mammals to the category “Vegetation.” We assigned mammals to the “Water” foraging stratus when the comment “Freshwater” was present in the field ForStra‐Comment or the species was known to forage or breed in paddy fields at the regional scale (Vigorita & Cucè, 2008).

We combined the diet and foraging classes by means of a full factorial design from which unrepresented combinations were excluded. In this way, we obtained 16 DFGs for birds and 15 DFGs for mammals (Tables 1, S4, and S5). We assigned a species to a DFG if the relative importance for both the corresponding diet and foraging stratus category were equal to or higher than 20%. This criterion follows a conservative worst‐case approach, whereby we considered that a species fully (100%) forages on every resource composing its diet, even though the relative proportion of food categories consumption is low; at the same time, the criterion excludes occasionally fed resources (<20%). The application of this approach leads to the possible inclusion of a species in multiple guilds, except for mammals that were exclusively assigned to a foraging stratus in the EltonTraits database (Tables S4 and S5). For birds, we checked species traits against literature data (Birds of the Western Palearctic Interactive 2.0.1© BirdGuides Ltd. 2004–2006) and modified DFG assignment when the standardized interpretation of the EltonTraits database did not ensure the application of a worst‐case approach.

**TABLE 1:** Focal species for DFG of birds and mammals in rice fields according to three modeling approaches

DFG		Birds				Mammals	
Diet	Stratus	*N*	EBM	SPM	SDM	*N*	EBM
**Invertebrates**	**Water**	**12**	**Great Reed‐warbler**	**Northern Lapwing**	**Black‐winged Stilt**	**4**	**Eurasian harvest mouse**
	Ground	28	Yellow Wagtail	Yellow Wagtail	Barn swallow	17	Etruscan Shrew
	Vegetation	22	Marsh warbler	Great Tit	Great Tit	7	‐
	**Aerial**	**9**	**Northern House‐martin**	**Northern House‐martin**	**Barn swallow**	**17**	**Lesser horseshoe bat**
**Vertebrates**	**Water**	**7**	**Squacco Heron**	**Little Egret**	**Little Egret**	**2**	**Eurasian water shrew**
	Ground	12	Great Reed‐warbler	Black‐billed Magpie	Common Kestrel	8	Least Weasel
	Aerial	3	Common Kestrel	Eurasian Golden Oriole	Eurasian Golden Oriole	‐	‐
	Vegetation	‐	‐	‐	‐	1	‐
**Fish**	**Water**	**7**	**Squacco Heron**	**Little Egret**	**Little Egret**	**4**	**Eurasian water shrew**
Carrions	Water	‐	‐	‐	‐	‐	‐
	Ground	2	Black‐billed Magpie	Black‐billed Magpie	Black‐billed Magpie	5	Apennine Shrew
Fruits	Vegetation	16	Marsh warbler	European Goldfinch	Great Tit	‐	‐
**Seeds**	**Water**	**2**	**Common Moorhen**	**Common Moorhen**	**Common Moorhen**	**2**	**Eurasian harvest mouse**
	Ground	16	European Goldfinch	European Goldfinch	Eurasian Tree Sparrow	9	House Mouse
	Vegetation	15	Blue Tit	European Goldfinch	Great Tit	4	‐
**Plants**	**Water**	**2**	**Common Moorhen**	**Common Moorhen**	**Common Moorhen**	**4**	**Eurasian harvest mouse**
	Ground	11	European Goldfinch	European Goldfinch	Eurasian Chaffinch	18	House Mouse
	Vegetation	8	European Goldfinch	European Goldfinch	Eurasian Chaffinch	5	‐

*Note*: DFGs were derived after modifications (see Methods section for details) from diet and foraging stratus categories of the EltonTraits database (adapted from Wilman et al., 2014). A species can be included in one or more categories. In bold, relevant categories for Risk Assessment in rice fields are shown.

Abbreviations: DFG, diet‐foraging guild; N, the number of species owing to DFG; EBM, expert‐based model; SDM, species distribution model; SPM, spatial pattern model.

Among DFGs, we considered six guilds as relevant for RA in rice cultivations on the basis of consistency with the flooded rice environment. Benthophagous included species feeding on invertebrates in the water column or on the bottom of the paddy field (Lupi et al., 2013), which are directly exposed to pesticides (Suhling et al., 2000). Similarly, the guild of aerial insectivorous is exposed to pesticides, which can be accumulated by flying and foliar adult insects emerging from paddy fields during the life cycle. Carnivorous vertebrates can be exposed to pesticides by feeding on other vertebrates living in the water stratum. We distinguished piscivorous species, feeding exclusively on fish, and water carnivorous, encompassing birds and mammals feeding on vertebrates other than fish, that is amphibians and reptiles, or other birds and mammals. The guild of water herbivorous was considered relevant as birds and mammals may feed on rice or other aquatic macrophytes, treated by herbicides and insecticides. Although the deposition of seeds on soil in flooded fields can be irrelevant, we also considered the guild of water granivorous. These species can eat seeds from the water surface or from the soil accumulated after ploughing at the margin of paddies and within the field (Stafford et al., 2010), whose role in paddy ecosystems deserves deeper investigation (Elphick et al., 2010).

For each guild and for each frequency index, we filtered the starting list by removing those species with an average frequency in rice paddies lower than 0.1 to remove rare species or those that avoid paddies in the study area, while keeping specialized paddy‐dwelling species or common generalists. In the last step, we extracted from the filtered list the DFG focal species, that is, the species with the lowest body mass, according to a conservative worst‐case approach.

## RESULTS

### Birds

**FIGURE 4:**
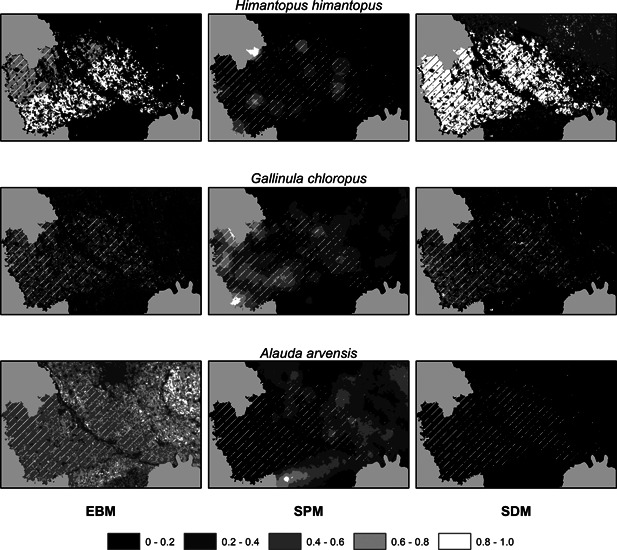
Example of spatial prediction maps in the western study area for an uncommon rice‐dwelling bird (upper row), a common rice‐dwelling bird (middle row) and a common rice‐avoiding bird (lower row). Maps were obtained by applying three modeling approaches (columns). EBM, expert‐based model; SDM, species distribution model; SPM, spatial pattern model. The plotted legend is valid for all maps, except for the SPM map of *Himantopus himantopus*, whose breaks are 0.05, 0.10, 0.15, and 0.20

We collected 41 866 observations in 3646 point counts (880 in or close to rice‐paddy fields) performed from 2000 to 2016 in the two study areas (Figure 1B). We observed 143 bird species, 121 of which (84.6%) were in rice fields. The number of detections per species in rice paddies ranged from 1 to 816 (Table S1). After removing rare species and excluding the Domestic Pigeon (*Columba livia domestica*), whose distribution is strictly dependent on human presence, we obtained a starting list composed of 48 species (33.6% of species richness of the study area). For each species, we produced three predictive maps applying EBM, SPM, and SDM (Figure 4).

**FIGURE 5:**
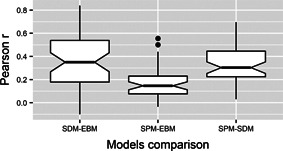
Pairwise comparisons of outputs of three modeling approaches. EBM, expert‐based model; SDM, species distribution model; SPM, spatial pattern model. Each boxplot shows the distribution of the Pearson correlation coefficients between the output maps of the involved model for 48 bird species

The mean suitability index in paddy fields estimated by EBM maps ranged from 0.018 to 0.775; the mean presence density in rice fields estimated by SPM maps ranged from 0.026 to 0.896; and the mean presence probability in paddy fields estimated by SDM maps ranged from 0.0014 to 0.878. The mean Pearson correlation coefficient between SDM and SPM maps was 0.329 (±0.165 SD), 0.353 (±0.231 SD) between SDM and EBM maps, and 0.170 (±0.139 SD) between SPM and EBM maps (Figure 5).

The number of species per DFG ranged from 2 to 28 (Table 1). After filtering species lists by frequency in rice fields, we identified the lightest species for each DFG and each modeling approach. Among water insectivores, the Black‐winged Stilt (*Himantopus himantopus*) was selected as focal species by SDM, while the Northern Lapwing (*V. vanellus*) was selected by SPM and the Great Reed‐warbler (*Acrocephalus arundinaceus*) by the EBM approach. The Barn swallow (*Hirundo rustica*) resulted the focal species for aerial insectivores according to SDM, while the Northern House‐martin (*Delichon urbica*) was identified as focal species by both SPM and EBM approach. Among water carnivores, SDM and SPM were consistent in identifying the Little Egret (*Egretta garzetta*), while EBM led to identification of Squacco Heron (*Ardeola ralloides*). Similarly, Little Egret was the focal species of piscivorous birds according to SDM and SPM, while Squacco Heron was selected by the EBM approach. The Common Moorhen (*G. chloropus*) was consistently identified by the three approaches for both the DFG of water granivorous and water herbivorous birds. Focal species for DFGs not relevant to RA in rice cultivations are reported in Table 1.

### Mammals

The starting list of rice‐dwelling mammals possibly occurring in the study area was composed of 65 species (73.0% of the mammal fauna of Lombardy), including native and naturalized species. Species frequency inside paddy fields, that is, the EBM suitability index, ranged from 0.002 to 0.938 (Table S5).

The number of mammals per DFG ranged from 1 to 18 (Table 1). After filtering species lists by frequency in rice fields, we identified the lightest species for each DFG. The Eurasian harvest mouse (*Micromys minutus*) was selected as focal species for invertebrate, seed and plant DFGs of the water stratus. The Eurasian Water Shrew (*Neomys fodiens*) was the focal species for aquatic vertebrates and fish. Water scavengers did not yield a representative species in the study area. We identified the Lesser horseshoe bat (*Rhinolophus hipposideros*) as focal species for aerial insectivorous mammals. The focal species for the remaining DFGs are shown in Table 1.

## DISCUSSION

The EFSA GDs for RA required by the European Pesticide Registration were developed on terrestrial crops. Guidance documents estimate exposure by identifying species that maximize the Food Intake Rate per body weight. However, the feeding guilds and the species of birds and mammals proposed by EFSA GD are inconsistent for rice cultivations, which are managed by flooding during the growing season. Vallon et al. (2018) tentatively filled this gap by a meta‐analysis of studies performed in Spain and France but stated the need for quantitative studies to identify focal species for higher‐tier RA, as indicated in the EFSA GD. We followed up the call of Vallon and coauthors and proposed an analytical framework that can be adapted to multiple sources of data across European rice‐producing countries to produce national lists of focal species for higher‐tier RA and potentially to any other crop type. We tested the method in a representative area of the largest rice‐cultivated region of Europe (northern Italy), which was not included in Vallon et al. (2018). Italy is the main European rice producer within the continental biogeographic region, while all other countries (except for Romania) are in the Mediterranean region. Italian rice cultivations are at the border between cohesive subregions of bird and mammal communities (Rueda et al., 2010). This particular biogeographic location can lead to divergent baseline communities (Newton, 2004) and composition of foraging guilds (Kissling et al., 2012), which can affect the species identified for RA.

Another source of dissimilarity between our lists and those proposed by Vallon and coauthors is the definition and composition of DFGs, with the main difference lying in the application of a worst‐case approach. We applied the approach by including a species in a DFG when food resources are used even scarcely by the species, so that a species can concur to focal species selection in multiple DFGs (note that this is different from the worst‐case concept adopted in RA). Conversely, Vallon and coauthors assigned a species to its only or main diet guild and they did not distinguish between foraging strata. We argue for the application of a worst‐case approach that guarantees higher protection for species of conservation concern. A direct consequence of the application of such a worst‐case approach to DFGs is the removal of the omnivorous guild. Disentangling foraging strata allowed a better description of the feeding behavior of birds and mammals, which can be particularly complex in natural and agricultural aquatic ecosystems (Czech & Parsons, 2002; Pérez‐Crespo et al., 2013; Zakaria & Rajpar, 2010). Often, many species that do not breed inside rice fields can use specific foraging strata of rice fields for feeding, thus enlarging the candidate set for focal species selection. For example, in rice cultivations, the guild of insectivorous birds that pick invertebrates from water is well separated from the guild of birds feeding on aerial adults of semiaquatic insects, whose larvae could have been exposed to pesticides within the water stratum during their development (Kraus & Stout, 2019). Therefore, focal species of the former guild cannot cover the ecological requirements of the species belonging to the latter. In addition, we adopted a different definition of the piscivorous/carnivorous diet categories: in the piscivorous guild, we included all species feeding on fish only, while amphibian specialist predators were included in the carnivorous guild. This led to a wider representation of carnivores in rice cultivations with respect to Vallon et al. (2018).

According to European EFSA GD, focal species selection for higher‐tier RA should be performed on data collected by an ideal systematic survey inside fields of the target crop during the period of maximum exposure. However, such data are seldom available due to lack of human and economic resources. Here, we demonstrated how the quality of other sources of data and the analytical approach used to estimate species frequency could affect focal species selection.

We considered the SPM approach to be the closest to an ideal survey when the amount of data is very large. This method can provide reliable lists based on the actual distribution of species in the study area but excludes rare species, even if they are rice specialists. SPM results should be carefully generalized to other contexts where local differences in landscape composition and agricultural practices can shape different animal communities (Vallon et al., 2018). In contrast, the list obtained by SPM can be valuable for close areas, in our example, for the western Po valley, which is in contiguity with our study area.

Expert‐based models are the unique choice when no census data are available or only qualitative data are available. In such cases, the prediction of species frequency through a literature‐based model produced reliable lists of focal species in 50% of DFGs (3/6 categories agree between EBM and SPM, Table 1). The limitation of this method is the tendency to include focal species that are very rare, such as the Great Reed‐warbler and the Squacco Heron, in our study area. Rice cultivations are potentially suitable for these birds, but the realized distribution of species can be limited by different exogenous constraints, such as the scarcity of small‐scale habitats (e.g., reed belts), whose distribution was not included in the available land‐use maps and thus in the models. Extreme caution must be exercised when interpreting EBM results or extrapolating them to other contexts. The EBM approach is better suited for a preliminary investigation of a candidate set of focal species, which should be explored in depth by means of quantitative data in a second stage (Di Febbraro et al., 2018).

Species distribution models integrate SPM information by relating species occurrence to habitat availability, and they SDMs can include rare species, as EBM does, but their suitability is related to the actual distribution of the species in rice cultivations. In this way, only rare species that find suitable ecological conditions in the study area are selected. Among the three compared approaches, SDM results are the most generalizable to other areas where similar environmental conditions, including agricultural practices, are maintained. We recognize that the resulting lists could be affected by the choice of the detecting radius, used in both EBM and SDM. However, the choice of the 250 m radius was made to represent the land‐use cover that affects species occurrence according to the technique adopted in bird surveys.

Given the above, bird focal species can be selected from the SPM or SDM list according to the region where the RA procedure is to be applied. Among water insectivorous, the Black‐winged Stilt is more exposed to pesticides (the lightest from SDM), confirming its sensitivity to pesticide application (Toral et al., 2015). However, in our study area, it is patchily distributed (Figure 4), and thus very rare on average. In Italy, it can be substituted by the Northern Lapwing, which is locally more abundant. It should be noted that the Northern Lapwing is also the second lightest species in the SDM list, and the EBM approach reports the two species at the second and third rank. Since this result completely agrees with Vallon et al. (2018), the two species can be reliably proposed as representative insectivorous water birds in Europe, where they are widely distributed in rice‐producing countries (Keller et al., 2020). We identified the Little Egret as focal species for water vertebrates, including carnivores and piscivorous. On the one hand, this confirms that the two DFGs can be considered together in RA for rice cultivations, as proposed by Vallon et al. (2018); on the other hand, it contrasts with the findings of Vallon and coauthors, which identified the Little Bittern (*Ixobrychus minutus*) as focal species. The latter and the Squacco Heron identified by EBM are very rare species in our study area and have a scattered distribution in rice‐producing countries due to the large variability in the presence of natural elements within cultivations, such as reed belts (Keller et al., 2020). For this reason, we believe that the Little Egret can better serve as a valid focal species for most European rice‐producing countries. Finally, the Moorhen was consistently selected as focal species for herbivorous birds, including seed and plant feeders (Lardjane‐Hamiti et al., 2015). This was the most frequent and by far the lightest herbivorous species in our study area, where it cohabits with the Mallard (*Anas platyrhynchos*) proposed by Vallon and coauthors. Conversely, we recorded very few detections of the Black‐headed gull, which was considered as very frequent by Vallon and coauthors and has a comparable body weight. Given that both species can breed inside rice fields, we believe that they can both be used as focal species of herbivorous and/or omnivorous birds for RA in Europe according to local prevalence. In addition, we identified the Barn swallow and the Northern House‐martin as focal species for aerial insectivorous birds. These Hirundinidae are widely distributed in Southern Europe and can be reliably adopted as focal species in different countries according to the local distribution.

The identification of focal mammals for higher‐tier RA in rice cultivations is tricky because quantitative data on mammals' frequency in rice fields are lacking (Vallon et al., 2018). Moreover, the strong uneven composition of mammal communities across European countries restricts the selection of species valid at the continental scale (EMMA1, https://www.european-mammals.org/php/mapmaker.php). For example, we identified two small mammals, the Eurasian harvest mouse and the Eurasian water shrew, as focal species for water insectivorous and herbivorous and water carnivorous and piscivorous, respectively. Both these species are lacking from rice cultivations in southern Spain and they are patchily distributed in Greece, Macedonia, and Romania (https://www.european-mammals.org/php/showmap.php?latname=Micromys+minutus&latname2=Neomys+fodiens, accessed 15 March 2021). On the other hand, the species proposed by Vallon et al. (2018), that is, the European Water Vole and the Brown Rat, are widely distributed in Europe, but they are by far heavier than other small mammals. Similarly, the authors proposed the Eurasian Otter as focal species for piscivorous mammals, but this species is totally absent from rice‐cultivated lands in Italy. Among aerial mammals, we selected the Lesser horseshoe bat, which is present in all rice‐producing countries in Europe (https://www.european-mammals.org/php/showmap.php?latname=Rhinolophus+hipposideros&latname2=, accessed 15 March 2021), although the local distribution and feeding behavior of the species in rice paddies are unknown (but see Toffoli & Rughetti, 2017 for the congener *Rhinolophus ferrumequinum*). Recent studies on bat distribution and feeding behavior in rice fields suggested that bats could be promising focal species for RA due to their sensitivity to management practices and peculiar pesticide exposure (Toffoli & Rughetti, 2017, 2020), which is uncovered by current approaches (European Food Safety Authority [EFSA] Panel on Plant Protection Products and their Residues [PPR] et al., 2019). However, for this and other groups of mammals, focal species selection is strongly biased by the lack of extensive data on diet and feeding behavior within paddies (Butet & Delettre, 2011; EFSA PPR et al., 2019). Given this uncertainty, we believe that focal species for mammals should be defined separately for the main geographic regions of European rice producers. To achieve this goal, we join the call of Vallon et al. for more specific studies on mammals' distribution and diet in rice fields.

The focal species that we proposed for higher‐tier RA in rice cultivations were calibrated on the basis of the period of main exposure, when paddies are flooded, rice grows and pesticides are applied according to the most frequent practice of continuous flooding (Parsons et al., 2010). However, agro‐management practices of rice cultivation are rapidly changing, with increasing use of dry‐seeding with postponed flooding cultivation (Zampieri et al., 2019). Although the adoption of dry seeding seems favorable to rice production within a certain limit, it also determines a drastic change in the availability and timing of water habitats, with direct consequences on the composition of wildlife communities that inhabit rice paddies (Pires et al., 2015; Rossaro et al., 2014; Toral & Figuerola, 2010). Likely, ground insectivorous and granivorous animals will substitute water‐dwelling species during pesticide application (Nam et al., 2015), but we warn against a blind adoption of the guidance available for other cereals, since the turnover of animal communities can differ according to local and surrounding environmental characteristics (Zellweger et al., 2017) as well as management practices (Hendershot et al., 2020).

## CONCLUSION

In this study, we proposed a tiered analytical framework that can be adapted to multiple sources of wildlife and habitat data to define focal species for higher‐tier RA in the context of European guidance. The definition of which data and methods should be adopted to identify focal species for higher‐tier RA in Europe has been widely debated because the current guidance (EFSA GD) allows use of multiple sources of data, but suggests an analytical approach valid for ad hoc field data only. Our analytical framework extends the spectrum of analytical methods to large‐scale census data collected for other purposes (e.g., wildlife monitoring). We applied the method to fill the gap concerning exposure scenarios related to rice cultivations in the RA guidance for birds and mammals. Building models from detection data was proven to be effective for defining crop‐specific species that allow one to obtain focused and coherent results in the process of RA according to the target crop. We identified a set of species valid for Italy, the main European rice producer, but the method can be applied in other countries according to data availability and can be generalized to other crops. Given the strong variability in species distribution and agro‐management practices among countries (Ibáñez et al., 2010), the picture that is emerging for rice cultivation is one of a spatially and temporally flexible list of focal species (e.g., Dietzen et al., 2014). For this purpose, our and previous studies reported the need to collect quantitative data on birds and mammals in permanently flooded rice paddies and in dry‐seeded fields. Once European lists of focal species for higher‐tier RA are consolidated, these can be used to identify indicator species and general focal species for lower‐tier RA in a designated guidance for rice cultivations.

## CONFLICT OF INTEREST

The authors declare that there are no conflicts of interest.

## Supporting information

This article includes online‐only Supporting Information.

The Supporting Information file includes the list of breeding birds detected in the study area, the table of land use change in the study area, the list of land‐use classes used in analyses, the list of candidate breeding birds, the list of candidate mammals, the figure of Habitat suitability functions used in Expert‐Based Models.

## Data Availability

Data collected using Research Fund of the University of Milano‐Bicocca, associated metadata and calculation tools are available from corresponding author Valerio Orioli (valerio.orioli@unimib.it). Data collected on behalf of the Lombardy Regional Administration and associated metadata can be obtained from the Lombardy Regional Administration upon reasonable request (https://www.regione.lombardia.it/wps/portal/istituzionale/HP/DettaglioRedazionale/istituzione/direzioni-generali/direzione-generale-agricoltura-alimentazione-e-sistemi-verdi/organizzazione-e-uffici/red-uo-sviluppo-sistemi-forestali-agricoltura-montagna-uso-e-tutela-suolo-politiche-faunistico-venatorie-agr).
