# Supplementary material for: Quantitative selection of focal birds and mammals in higher‐tier risk assessment: An application to rice cultivations
Source: Integr Environ Assess Manag. 2021 Nov 3;18(4):1020–34. doi: 10.1002/ieam.4535 (PMC9298216; doi:10.1002/ieam.4535)
Supplement: Supplementary file 1 — The Supporting Information file includes the list of breeding birds detected in the study area, the table of land use change in the study area, the list of land‐use classes used in analyses, the list of candidate breeding birds, the list of candidate mammals, the figure of Habitat suitability functions used in Expert‐Based Models. [file IEAM-18-1020-s001.docx]

**Quantitative selection of focal birds and mammals in higher-tier risk assessment: an application to rice cultivations**

SUPPORTING INFORMATION

**Table S1**. List of breeding birds detected in the study area p.2

**Table S2**. Land use change in the study area p.5

**Table S3**. Land-use classes p.6

**Table S4**. List of candidate breeding birds p.7

**Table S5**. List of candidate mammals p.9

**Figure S1.** Habitat suitability functions used in Expert-Based Models p.11

**References** p.12

**Table S1**. List of breeding birds detected in the study area in 2000-2016. Taxonomy according to Storchová et al. (2018). Study_area: total number of occurrences in the study area; Rice_fields: number of occurrences in point counts in or close to rice cultivations (≥5% of rice cultivations in a buffer of 250 meters around the point); %Rice_fields: proportion of occurrences on the total number of point counts performed in or close to rice cultivations (n=880). Species with 25 (~2.5%) or more occurrences in rice fields are highlighted in grey.

| **Species** | **Order** | **Family** | **Euring_code** | **Study_area** | **Rice_fields** | **%Rice_fields** |
| --- | --- | --- | --- | --- | --- | --- |
| *Phalacrocorax carbo* | Suliformes | Phalacrocoracidae | E00720 | 141 | 56 | 6.36% |
| *Ixobrychus minutus* | Pelecaniformes | Ardeidae | E00980 | 15 | 10 | 1.14% |
| *Nycticorax nycticorax* | Pelecaniformes | Ardeidae | E01040 | 571 | 355 | 40.34% |
| *Ardeola ralloides* | Pelecaniformes | Ardeidae | E01080 | 33 | 27 | 3.07% |
| *Bubulcus ibis* | Pelecaniformes | Ardeidae | E01110 | 190 | 92 | 10.45% |
| *Egretta garzetta* | Pelecaniformes | Ardeidae | E01190 | 965 | 523 | 59.43% |
| *Ardea alba* | Pelecaniformes | Ardeidae | E01210 | 74 | 38 | 4.32% |
| *Ardea cinerea* | Pelecaniformes | Ardeidae | E01220 | 1283 | 485 | 55.11% |
| *Ardea purpurea* | Pelecaniformes | Ardeidae | E01240 | 156 | 71 | 8.07% |
| *Ciconia ciconia* | Ciconiiformes | Ciconiidae | E01340 | 34 | 14 | 1.59% |
| *Cygnus olor* | Anseriformes | Anatidae | E01520 | 5 | 1 | 0.11% |
| *Anas platyrhynchos* | Anseriformes | Anatidae | E01860 | 733 | 334 | 37.95% |
| *Pernis apivorus* | Accipitriformes | Accipitridae | E02310 | 19 | 3 | 0.34% |
| *Milvus migrans* | Accipitriformes | Accipitridae | E02380 | 39 | 1 | 0.11% |
| *Circus aeruginosus* | Accipitriformes | Accipitridae | E02600 | 41 | 10 | 1.14% |
| *Circus cyaneus* | Accipitriformes | Accipitridae | E02610 | 1 | 1 | 0.11% |
| *Circus pygargus* | Accipitriformes | Accipitridae | E02630 | 15 | 1 | 0.11% |
| *Accipiter gentilis* | Accipitriformes | Accipitridae | E02670 | 1 | 0 | <0.01% |
| *Accipiter nisus* | Accipitriformes | Accipitridae | E02690 | 22 | 1 | 0.11% |
| *Buteo buteo* | Accipitriformes | Accipitridae | E02870 | 160 | 57 | 6.48% |
| *Falco tinnunculus* | Falconiformes | Falconidae | E03040 | 343 | 63 | 7.16% |
| *Falco vespertinus* | Falconiformes | Falconidae | E03070 | 14 | 2 | 0.23% |
| *Falco subbuteo* | Falconiformes | Falconidae | E03100 | 112 | 29 | 3.30% |
| *Falco peregrinus* | Falconiformes | Falconidae | E03200 | 8 | 2 | 0.23% |
| *Colinus virginianus* | Galiiformes | Odontophoridae | E03450 | 9 | 4 | 0.45% |
| *Alectoris rufa* | Galiiformes | Phasianidae | E03580 | 9 | 0 | <0.01% |
| *Perdix perdix* | Galiiformes | Phasianidae | E03670 | 3 | 0 | <0.01% |
| *Coturnix coturnix* | Galiiformes | Phasianidae | E03700 | 165 | 11 | 1.25% |
| *Phasianus colchicus* | Galiiformes | Phasianidae | E03940 | 794 | 173 | 19.66% |
| *Rallus aquaticus* | Gruiformes | Rallidae | E04070 | 1 | 0 | <0.01% |
| *Gallinula chloropus* | Gruiformes | Rallidae | E04240 | 659 | 323 | 36.70% |
| *Fulica atra* | Gruiformes | Rallidae | E04290 | 23 | 7 | 0.80% |
| *Himantopus himantopus* | Charadriiformes | Recurvirostridae | E04550 | 45 | 39 | 4.43% |
| *Charadrius dubius* | Charadriiformes | Charadriidae | E04690 | 28 | 1 | 0.11% |
| *Charadrius hiaticula* | Charadriiformes | Charadriidae | E04700 | 1 | 1 | 0.11% |
| *Pluvialis squatarola* | Charadriiformes | Charadriidae | E04860 | 1 | 1 | 0.11% |
| *Vanellus vanellus* | Charadriiformes | Charadriidae | E04930 | 325 | 258 | 29.32% |
| *Calidris minuta* | Charadriiformes | Scolopacidae | E05010 | 2 | 2 | 0.23% |
| *Calidris ferruginea* | Charadriiformes | Scolopacidae | E05090 | 1 | 1 | 0.11% |
| *Calidris pugnax* | Charadriiformes | Scolopacidae | E05170 | 2 | 2 | 0.23% |
| *Numenius arquata* | Charadriiformes | Scolopacidae | E05410 | 1 | 1 | 0.11% |
| *Tringa totanus* | Charadriiformes | Scolopacidae | E05460 | 2 | 1 | 0.11% |
| *Tringa nebularia* | Charadriiformes | Scolopacidae | E05480 | 6 | 4 | 0.45% |
| *Tringa ochropus* | Charadriiformes | Scolopacidae | E05530 | 8 | 4 | 0.45% |
| *Tringa glareola* | Charadriiformes | Scolopacidae | E05540 | 5 | 5 | 0.57% |
| *Actitis hypoleucos* | Charadriiformes | Scolopacidae | E05560 | 9 | 1 | 0.11% |
| *Larus ridibundus* | Charadriiformes | Laridae | E05820 | 23 | 9 | 1.02% |

| **Species** | **Order** | **Family** | **Euring_code** | **Study_area** | **Rice_fields** | **%Rice_fields** |
| --- | --- | --- | --- | --- | --- | --- |
| *Larus argentatus* | Charadriiformes | Laridae | E05920 | 14 | 3 | 0.34% |
| *Larus michahellis* | Charadriiformes | Laridae | E05926 | 67 | 12 | 1.36% |
| *Sterna hirundo* | Charadriiformes | Laridae | E06150 | 44 | 16 | 1.82% |
| *Sternula albifrons* | Charadriiformes | Laridae | E06240 | 12 | 3 | 0.34% |
| *Chlidonias hybrida* | Charadriiformes | Laridae | E06260 | 1 | 0 | <0.01% |
| *Chlidonias niger* | Charadriiformes | Laridae | E06270 | 2 | 1 | 0.11% |
| *Columba livia domestica* | Columbiformes | Columbidae | E06650 | 1465 | 361 | 41.02% |
| *Columba oenas* | Columbiformes | Columbidae | E06680 | 4 | 2 | 0.23% |
| *Columba palumbus* | Columbiformes | Columbidae | E06700 | 878 | 170 | 19.32% |
| *Streptopelia decaocto* | Columbiformes | Columbidae | E06840 | 1645 | 272 | 30.91% |
| *Streptopelia turtur* | Columbiformes | Columbidae | E06870 | 400 | 80 | 9.09% |
| *Psittacula krameri* | Psittaciformes | Psittacidae | E07120 | 6 | 1 | 0.11% |
| *Cuculus canorus* | Cuculiformes | Cuculidae | E07240 | 740 | 225 | 25.57% |
| *Tyto alba* | Strigiformes | Tytonidae | E07350 | 1 | 0 | <0.01% |
| *Otus scops* | Strigiformes | Strigidae | E07390 | 1 | 0 | <0.01% |
| *Athene noctua* | Strigiformes | Strigidae | E07570 | 69 | 10 | 1.14% |
| *Strix aluco* | Strigiformes | Strigidae | E07610 | 5 | 1 | 0.11% |
| *Asio otus* | Strigiformes | Strigidae | E07670 | 3 | 2 | 0.23% |
| *Caprimulgus europaeus* | Caprimulgiformes | Caprimulgidae | E07780 | 2 | 0 | <0.01% |
| *Apus apus* | Caprimulgiformes | Apodidae | E07950 | 1272 | 269 | 30.57% |
| *Apus pallidus* | Caprimulgiformes | Apodidae | E07960 | 1 | 0 | <0.01% |
| *Tachymarptis melba* | Caprimulgiformes | Apodidae | E07980 | 2 | 0 | <0.01% |
| *Alcedo atthis* | Coraciiformes | Alcedinidae | E08310 | 55 | 9 | 1.02% |
| *Merops apiaster* | Coraciiformes | Meropidae | E08400 | 125 | 16 | 1.82% |
| *Upupa epops* | Coraciiformes | Upupidae | E08460 | 49 | 19 | 2.16% |
| *Jynx torquilla* | Piciformes | Picidae | E08480 | 37 | 5 | 0.57% |
| *Picus viridis* | Piciformes | Picidae | E08560 | 194 | 36 | 4.09% |
| *Dendrocopos major* | Piciformes | Picidae | E08760 | 408 | 74 | 8.41% |
| *Dryobates minor* | Piciformes | Picidae | E08870 | 4 | 2 | 0.23% |
| *Calandrella brachydactyla* | Passeriformes | Alaudidae | E09680 | 3 | 1 | 0.11% |
| *Galerida cristata* | Passeriformes | Alaudidae | E09720 | 60 | 2 | 0.23% |
| *Lullula arborea* | Passeriformes | Alaudidae | E09740 | 3 | 0 | <0.01% |
| *Alauda arvensis* | Passeriformes | Alaudidae | E09760 | 660 | 66 | 7.50% |
| *Riparia riparia* | Passeriformes | Hirundinidae | E09810 | 4 | 1 | 0.11% |
| *Hirundo rustica* | Passeriformes | Hirundinidae | E09920 | 2054 | 425 | 48.30% |
| *Delichon urbicum* | Passeriformes | Hirundinidae | E10010 | 521 | 72 | 8.18% |
| *Anthus campestris* | Passeriformes | Motacillidae | E10050 | 1 | 1 | 0.11% |
| *Motacilla flava* | Passeriformes | Motacillidae | E10170 | 731 | 126 | 14.32% |
| *Motacilla cinerea* | Passeriformes | Motacillidae | E10190 | 9 | 4 | 0.45% |
| *Motacilla alba* | Passeriformes | Motacillidae | E10200 | 166 | 34 | 3.86% |
| *Troglodytes troglodytes* | Passeriformes | Troglodytidae | E10660 | 57 | 14 | 1.59% |
| *Prunella modularis* | Passeriformes | Prunellidae | E10840 | 1 | 0 | <0.01% |
| *Erithacus rubecula* | Passeriformes | Muscicapidae | E10990 | 24 | 3 | 0.34% |
| *Luscinia megarhynchos* | Passeriformes | Muscicapidae | E11040 | 2033 | 518 | 58.86% |
| *Phoenicurus ochruros* | Passeriformes | Muscicapidae | E11210 | 16 | 1 | 0.11% |
| *Phoenicurus phoenicurus* | Passeriformes | Muscicapidae | E11220 | 60 | 3 | 0.34% |
| *Saxicola rubetra* | Passeriformes | Muscicapidae | E11370 | 6 | 1 | 0.11% |
| *Saxicola torquatus* | Passeriformes | Muscicapidae | E11390 | 120 | 19 | 2.16% |
| *Oenanthe oenanthe* | Passeriformes | Muscicapidae | E11460 | 2 | 0 | <0.01% |
| *Turdus torquatus* | Passeriformes | Turdidae | E11860 | 1 | 0 | <0.01% |
| *Turdus merula* | Passeriformes | Turdidae | E11870 | 1996 | 381 | 43.30% |
| *Turdus pilaris* | Passeriformes | Turdidae | E11980 | 1 | 0 | <0.01% |
| *Cettia cetti* | Passeriformes | Scotocercidae | E12200 | 279 | 61 | 6.93% |

| **Species** | **Order** | **Family** | **Euring_code** | **Study_area** | **Rice_fields** | **%Rice_fields** |
| --- | --- | --- | --- | --- | --- | --- |
| *Cisticola juncidis* | Passeriformes | Cisticolidae | E12260 | 44 | 1 | 0.11% |
| *Locustella luscinioides* | Passeriformes | Locustellidae | E12380 | 2 | 2 | 0.23% |
| *Acrocephalus schoenobaenus* | Passeriformes | Acrocephalidae | E12430 | 1 | 0 | <0.01% |
| *Acrocephalus palustris* | Passeriformes | Acrocephalidae | E12500 | 104 | 46 | 5.23% |
| *Acrocephalus scirpaceus* | Passeriformes | Acrocephalidae | E12510 | 42 | 14 | 1.59% |
| *Acrocephalus arundinaceus* | Passeriformes | Acrocephalidae | E12530 | 69 | 25 | 2.84% |
| *Hippolais polyglotta* | Passeriformes | Acrocephalidae | E12600 | 146 | 27 | 3.07% |
| *Sylvia cantillans* | Passeriformes | Sylviidae | E12650 | 1 | 0 | <0.01% |
| *Sylvia communis* | Passeriformes | Sylviidae | E12750 | 51 | 2 | 0.23% |
| *Sylvia borin* | Passeriformes | Sylviidae | E12760 | 2 | 1 | 0.11% |
| *Sylvia atricapilla* | Passeriformes | Sylviidae | E12770 | 2230 | 558 | 63.41% |
| *Phylloscopus sibilatrix* | Passeriformes | Phylloscopidae | E13080 | 8 | 3 | 0.34% |
| *Phylloscopus collybita* | Passeriformes | Phylloscopidae | E13110 | 10 | 0 | <0.01% |
| *Muscicapa striata* | Passeriformes | Muscicapidae | E13350 | 214 | 34 | 3.86% |
| *Panurus biarmicus* | Passeriformes | Panuridae | E13640 | 1 | 1 | 0.11% |
| *Aegithalos caudatus* | Passeriformes | Aegithalidae | E14370 | 140 | 26 | 2.95% |
| *Poecile palustris* | Passeriformes | Paridae | E14400 | 10 | 3 | 0.34% |
| *Periparus ater* | Passeriformes | Paridae | E14610 | 1 | 0 | <0.01% |
| *Cyanistes caeruleus* | Passeriformes | Paridae | E14620 | 199 | 31 | 3.52% |
| *Parus major* | Passeriformes | Paridae | E14640 | 1397 | 291 | 33.07% |
| *Sitta europaea* | Passeriformes | Sittadae | E14790 | 14 | 2 | 0.23% |
| *Certhia brachydactyla* | Passeriformes | Certhiidae | E14870 | 1 | 0 | <0.01% |
| *Remiz pendulinus* | Passeriformes | Remizidae | E14900 | 11 | 0 | <0.01% |
| *Oriolus oriolus* | Passeriformes | Oriolidae | E15080 | 472 | 119 | 13.52% |
| *Lanius collurio* | Passeriformes | Laniidae | E15150 | 87 | 16 | 1.82% |
| *Garrulus glandarius* | Passeriformes | Corvidae | E15390 | 124 | 18 | 2.05% |
| *Pica pica* | Passeriformes | Corvidae | E15490 | 840 | 95 | 10.80% |
| *Corvus monedula* | Passeriformes | Corvidae | E15600 | 36 | 3 | 0.34% |
| *Corvus corone* | Passeriformes | Corvidae | E15671 | 4 | 1 | 0.11% |
| *Corvus cornix* | Passeriformes | Corvidae | E15673 | 3019 | 816 | 92.73% |
| *Sturnus vulgaris* | Passeriformes | Sturnidae | E15820 | 2757 | 580 | 65.91% |
| *Passer italiae* | Passeriformes | Passeridae | E15912 | 2224 | 480 | 54.55% |
| *Passer montanus* | Passeriformes | Passeridae | E15980 | 1553 | 429 | 48.75% |
| *Fringilla coelebs* | Passeriformes | Fringillidae | E16360 | 871 | 146 | 16.59% |
| *Serinus serinus* | Passeriformes | Fringillidae | E16400 | 264 | 11 | 1.25% |
| *Chloris chloris* | Passeriformes | Fringillidae | E16490 | 478 | 54 | 6.14% |
| *Carduelis carduelis* | Passeriformes | Fringillidae | E16530 | 655 | 105 | 11.93% |
| *Linaria cannabina* | Passeriformes | Fringillidae | E16600 | 2 | 0 | <0.01% |
| *Emberiza citrinella* | Passeriformes | Emberizidae | E18570 | 3 | 0 | <0.01% |
| *Emberiza cirlus* | Passeriformes | Emberizidae | E18580 | 12 | 1 | 0.11% |
| *Emberiza hortulana* | Passeriformes | Emberizidae | E18660 | 7 | 1 | 0.11% |
| *Emberiza schoeniclus* | Passeriformes | Emberizidae | E18770 | 5 | 2 | 0.23% |
| *Emberiza calandra* | Passeriformes | Emberizidae | E18820 | 40 | 0 | <0.01% |

**Table S2**. Land use change in the study area between 1999 and 2015. Changes were measured in km^2^ by using Lombardy land-use maps for 1999 (DUSAF 1.1) and 2015 (DUSAF 5.0).

| **Land use** | **1999** | **2015** | **Δ** |
| --- | --- | --- | --- |
| Urban areas | 930 | 1099 | 18.2% |
| Arable lands | 3380 | 3200 | -5.3% |
| Rice cultivations | 1234 | 1041 | -15.7% |
| Permanent crops | 429 | 399 | -7.1% |
| Forage and fodder crops | 162 | 360 | 122.7% |
| Woodlands and Natural areas | 284 | 311 | 9.3% |
| Water bodies | 154 | 164 | 6.4% |

**Table S3**. Land-use classes obtained from Lombardy land-use map for 2015 (DUSAF5). Mean and standard deviation of class fractional cover measured in the field in a buffer of 250m around point counts are reported (input data for SDM).

| **Land-use class** | **Description** | **Mean (sd)** |
| --- | --- | --- |
| Dense urban areas | Dense residential areas, industrial and commercial areas, infrastructures | 18.61 (26.20) |
| Loose urban areas | Loose residential areas | 7.11 (12.29) |
| Set-aside | Set-aside, uncultivated fields | 2.61 (8.21) |
| Maize | Maize fields | 19.68 (27.17) |
| Other cereals | Wheat, barley, rye fields | 8.42 (18.02) |
| Other crops | Plowed fields, weeded fields, soy, covered greenhouses, vegetable gardens, small heterogeneous fields | 9.41 (19.02) |
| Nurseries | Outdoor plant nurseries | 0.156 (1.97) |
| Rice | Rice paddy fields | 12.60 (25.81) |
| Vineyards | Vineyards | 1.07 (7.44) |
| Orchards | Orchards | 0.380 (3.20) |
| Olive groves | Olive groves | 0.019 (0.536) |
| Poplar groves | Poplar groves | 3.68 (10.66) |
| Forage/fodder crops | Lowland forage or fodder crops | 9.60 (18.0) |
| Deciduous woodlands | Deciduous woodlands | 8.91 (15.91) |
| Coniferous woodlands | Coniferous woodlands | 0.083 (2.28) |
| Mixed woodlands | Woodlands of mixed deciduous and coniferous species | 0.113 (1.66) |
| Natural prairies | Natural prairies | 0.054 (1.20) |
| Shrublands | Shrubs, bank vegetation, transitional shrub/woodland | 0.671 (3.50) |
| Scarce vegetation | Open areas with scarce vegetation and rocks | 0.271 (2.64) |
| Wetlands | Palustrine wetlands and peatlands | 0.461 (2.92) |
| Lotic waters | Large rivers and artificial channels | 2.78 (7.26) |
| Lotic waters PA | Presence/absence of secondary lotic waters, small rivers, streams and irrigation ditches | 0.493 (0.50) |
| Lentic waters | Lakes, ponds and artificial reservoirs | 0.516 (4.37) |

**Table S4**. List of candidate breeding birds for focal species selection in rice cultivations. Frequency inside rice fields resulted from three approaches: SDM, Species Distribution Model; SPM, Spatial Point Model; EBM, Expert-based Model. BM: body mass in grams. Assignment of species to 16 Diet-Foraging Guilds (DFGs) according to diet (Inv, invertebrates’ eaters; Vert, carnivorous species, excluding fish; Fish, piscivorous species; Scav, scavengers, Frug, frugivorous species; Seed, seeds’ eaters; Herb, plants’ eaters) and foraging stratum (w, water; g, ground; v, vegetation; a, air).

| **Species** | **SDM** | **SPM** | **EBM** | **BM** | **Inv_w** | **Inv_g** | **Inv_v** | **Inv_a** | **Vert_w** | **Vert_g** | **Vert_a** | **Fish** | **Scav_g** | **Frug_v** | **Seed_w** | **Seed_g** | **Seed_v** | **Herb_w** | **Herb_g** | **Herb_v** |
| --- | --- | --- | --- | --- | --- | --- | --- | --- | --- | --- | --- | --- | --- | --- | --- | --- | --- | --- | --- | --- |
| *Phalacrocorax carbo* | 0.165 | 0.053 | 0.160 | 2528.97 | - | - | - | - | - | - | - | X | - | - | - | - | - | - | - | - |
| *Nycticorax nycticorax* | 0.367 | 0.364 | 0.334 | 810.00 | X | X | - | - | X | X | - | X | - | - | - | - | - | - | - | - |
| *Ardeola ralloides* | 0.035 | 0.026 | 0.431 | 287.00 | X | X | - | - | X | X | - | X | - | - | - | - | - | - | - | - |
| *Bubulcus ibis* | 0.353 | 0.086 | 0.568 | 365.95 | X | X | - | - | X | X | - | - | - | - | - | - | - | - | - | - |
| *Egretta garzetta* | 0.612 | 0.483 | 0.312 | 312.00 | X | X | - | - | X | X | - | X | - | - | - | - | - | - | - | - |
| *Ardea alba* | 0.103 | 0.033 | 0.361 | 871.33 | X | X | - | - | X | X | - | X | - | - | - | - | - | - | - | - |
| *Ardea cinerea* | 0.581 | 0.504 | 0.292 | 1443.00 | X | X | - | - | X | X | - | X | - | - | - | - | - | - | - | - |
| *Ardea purpurea* | 0.066 | 0.062 | 0.775 | 1064.48 | X | X | - | - | X | X | - | X | - | - | - | - | - | - | - | - |
| *Anas platyrhynchos* | 0.495 | 0.288 | 0.266 | 843.42 | X | X | - | - | - | - | - | - | - | - | X | X | - | X | X | - |
| *Buteo buteo* | 0.024 | 0.060 | 0.229 | 759.10 | - | - | - | - | - | X | - | - | - | - | - | - | - | - | - | - |
| *Falco tinnunculus* | 0.104 | 0.082 | 0.272 | 183.21 | - | - | - | - | - | X | X | - | - | - | - | - | - | - | - | - |
| *Falco subbuteo* | 0.035 | 0.040 | 0.208 | 208.17 | - | - | - | X | - | - | X | - | - | - | - | - | - | - | - | - |
| *Phasianus colchicus* | 0.157 | 0.186 | 0.167 | 1120.31 | - | X | - | - | - | - | - | - | - | - | - | X | X | - | X | X |
| *Gallinula chloropus* | 0.303 | 0.343 | 0.358 | 339.63 | X | X | X | - | - | - | - | - | - | X | X | X | X | X | X | X |
| *Himantopus himantopus* | 0.878 | 0.027 | 0.699 | 176.82 | X | - | - | X | - | - | - | - | - | - | - | - | - | - | - | - |
| *Vanellus vanellus* | 0.543 | 0.191 | 0.719 | 218.37 | X | X | - | - | - | - | - | - | - | - | - | - | - | - | - | - |
| *Columba palumbus* | 0.305 | 0.197 | 0.211 | 490.00 | - | - | - | - | - | - | - | - | - | X | - | X | X | - | X | X |
| *Streptopelia decaocto* | 0.172 | 0.364 | 0.142 | 148.96 | - | - | - | - | - | - | - | - | - | X | - | X | X | - | X | X |
| *Streptopelia turtur* | 0.091 | 0.095 | 0.226 | 132.00 | - | - | - | - | - | - | - | - | - | X | - | X | - | - | - | - |
| *Cuculus canorus* | 0.231 | 0.253 | 0.143 | 111.36 | - | X | X | - | - | - | - | - | - | - | - | - | - | - | - | - |
| *Apus apus* | 0.204 | 0.380 | 0.171 | 37.60 | - | - | - | X | - | - | - | - | - | - | - | - | - | - | - | - |
| *Picus viridis* | 0.025 | 0.059 | 0.084 | 176.00 | - | X | X | - | - | - | - | - | - | - | - | - | - | - | - | - |
| *Dendrocopos major* | 0.099 | 0.118 | 0.059 | 74.94 | - | - | X | - | - | - | - | - | - | - | - | - | X | - | - | - |
| *Alauda arvensis* | 0.024 | 0.110 | 0.471 | 37.31 | - | X | - | - | - | - | - | - | - | - | - | X | X | - | X | X |
| *Hirundo rustica* | 0.324 | 0.502 | 0.205 | 17.91 | - | X | X | X | - | - | - | - | - | - | - | - | - | - | - | - |
| *Delichon urbicum* | 0.034 | 0.104 | 0.177 | 14.50 | - | - | - | X | - | - | - | - | - | - | - | - | - | - | - | - |
| *Motacilla flava* | 0.033 | 0.139 | 0.463 | 17.68 | - | X | - | X | - | - | - | - | - | - | - | - | - | - | - | - |
| *Motacilla alba* | 0.050 | 0.047 | 0.151 | 23.93 | - | X | - | X | - | - | - | - | - | - | - | - | - | - | - | - |
| *Luscinia megarhynchos* | 0.388 | 0.581 | 0.102 | 19.60 | - | X | X | - | - | - | - | - | - | X | - | - | - | - | - | - |
| *Turdus merula* | 0.321 | 0.485 | 0.130 | 102.73 | - | X | X | - | - | - | - | - | - | X | - | X | X | - | - | - |
| *Cettia cetti* | 0.026 | 0.084 | 0.018 | 13.22 | - | X | X | - | - | - | - | - | - | - | - | - | - | - | - | - |
| *Acrocephalus palustris* | 0.030 | 0.037 | 0.216 | 11.50 | - | - | X | - | - | - | - | - | - | X | - | - | - | - | - | - |
| *Acrocephalus arundinaceus* | 0.001 | 0.026 | 0.118 | 30.00 | X | X | X | - | - | X | - | - | - | - | - | - | - | - | - | - |
| *Hippolais polyglotta* | 0.006 | 0.039 | 0.044 | 11.00 | - | - | X | - | - | - | - | - | - | X | - | - | - | - | - | - |
| *Sylvia atricapilla* | 0.696 | 0.624 | 0.093 | 16.70 | - | - | X | - | - | - | - | - | - | X | - | - | - | - | - | - |
| *Muscicapa striata* | 0.023 | 0.054 | 0.071 | 15.90 | - | X | X | X | - | - | - | - | - | X | - | - | - | - | - | - |
| *Aegithalos caudatus* | 0.021 | 0.035 | 0.025 | 8.60 | - | - | X | - | - | - | - | - | - | X | - | - | - | - | - | - |
| *Cyanistes caeruleus* | 0.007 | 0.053 | 0.248 | 13.30 | - | - | X | - | - | - | - | - | - | X | - | - | X | - | - | - |
| *Parus major* | 0.204 | 0.331 | 0.091 | 16.25 | - | - | X | - | - | - | - | - | - | X | - | - | X | - | - | - |
| *Oriolus oriolus* | 0.152 | 0.153 | 0.038 | 79.00 | - | - | X | X | - | - | X | - | - | X | - | - | - | - | - | - |
| *Pica pica* | 0.162 | 0.152 | 0.208 | 217.48 | - | X | X | - | - | X | - | - | X | - | - | X | - | - | X | - |
| *Corvus corone cornix* | 0.456 | 0.896 | 0.181 | 570.00 | - | X | - | - | - | X | - | - | X | - | - | X | - | - | X | - |
| *Sturnus vulgaris* | 0.401 | 0.674 | 0.205 | 77.14 | - | X | X | - | - | - | - | - | - | X | - | X | X | - | - | - |
| *Passer italiae* | 0.171 | 0.649 | 0.198 | 26.51 | - | X | X | - | - | - | - | - | - | - | - | X | X | - | X | X |
| *Passer montanus* | 0.237 | 0.446 | 0.225 | 21.39 | - | X | X | - | - | - | - | - | - | - | - | X | X | - | - | - |
| *Fringilla coelebs* | 0.101 | 0.190 | 0.038 | 23.81 | - | X | X | - | - | - | - | - | - | - | - | X | X | - | X | X |
| *Chloris chloris* | 0.018 | 0.096 | 0.188 | 26.00 | - | - | - | - | - | - | - | - | - | - | - | X | X | - | - | - |
| *Carduelis carduelis* | 0.047 | 0.181 | 0.157 | 16.00 | - | - | - | - | - | - | - | - | - | X | - | X | X | - | X | X |

**Table S5**. List of candidate mammals for focal species selection in rice cultivations. EBM: frequency inside rice fields resulted from the Expert-based Model. BM: body mass in grams. Assignment of species to 15 Diet-Foraging Guilds (DFGs) according to diet (I, invertebrates’ eaters; V, carnivorous species, excluding fish; F, piscivorous species; Sc, scavengers; Se, seeds’ eaters; H, plants’ eaters) and foraging stratum (w, water; g, ground; v, vegetation; a, air).

| **Species** | **EBM** | **BM** | **I_w** | **I_g** | **I_v** | **I_a** | **V_w** | **V_g** | **V_a** | **F** | **Sc_g** | **Se_w** | **Se_g** | **Se_v** | **H_w** | **H_g** | **H_v** |
| --- | --- | --- | --- | --- | --- | --- | --- | --- | --- | --- | --- | --- | --- | --- | --- | --- | --- |
| *Sciurus carolinensis* | 0.015 | 506.5 | - | - | - | - | - | - | - | - | - | - | - | X | - | - | X |
| *Sciurus vulgaris* | 0.072 | 333.0 | - | - | - | - | - | - | - | - | - | - | - | X | - | - | X |
| *Eliomys quercinus* | 0.006 | 115.0 | - | - | X | - | - | - | X | - | - | - | - | X | - | - | X |
| *Muscardinus avellanarius* | 0.033 | 27.5 | - | - | - | - | - | - | - | - | - | - | - | - | - | - | X |
| *Glis glis* | 0.015 | 128.1 | - | - | - | - | - | - | - | - | - | - | - | X | - | - | X |
| *Microtus arvalis* | 0.071 | 28.0 | - | - | - | - | - | - | - | - | - | - | X | - | - | X | - |
| *Microtus savii* | 0.132 | 20.0 | - | - | - | - | - | - | - | - | - | - | X | - | - | X | - |
| *Microtus multiplex* | 0.007 | 23.2 | - | - | - | - | - | - | - | - | - | - | X | - | - | X | - |
| *Arvicola amphibius* | 0.015 | 120.0 | - | - | - | - | - | - | - | - | - | - | - | - | X | - | - |
| *Myodes glareolus* | 0.479 | 20.7 | - | X | - | - | - | - | - | - | - | - | X | - | - | X | - |
| *Apodemus agrarius* | 0.191 | 23.3 | - | X | - | - | - | - | - | - | - | - | X | - | - | X | - |
| *Apodemus flavicollis* | 0.053 | 26.7 | - | X | - | - | - | - | - | - | - | - | X | - | - | X | - |
| *Apodemus sylvaticus* | 0.265 | 30.5 | - | X | - | - | - | - | - | - | - | - | X | - | - | X | - |
| *Micromys minutus* | 0.343 | 6.0 | X | - | - | - | - | - | - | - | - | X | - | - | X | - | - |
| *Mus musculus* | 0.189 | 16.3 | - | X | - | - | - | - | - | - | - | - | X | - | - | X | - |
| *Rattus norvegicus* | 0.607 | 338.3 | X | - | - | - | - | - | - | - | - | X | - | - | X | - | - |
| *Rattus rattus* | 0.588 | 229.1 | - | X | - | - | - | - | - | - | - | - | X | - | - | X | - |
| *Hystrix cristata* | 0.307 | 16250.0 | - | - | - | - | - | - | - | - | - | - | - | - | - | X | - |
| *Myocastor coypus* | 0.741 | 6937.5 | - | - | - | - | - | - | - | - | - | - | - | - | X | - | - |
| *Lepus europaeus* | 0.602 | 3740.0 | - | - | - | - | - | - | - | - | - | - | - | - | - | X | - |
| *Sylvilagus floridanus* | 0.281 | 1172.8 | - | - | - | - | - | - | - | - | - | - | - | - | - | X | - |
| *Oryctolagus cuniculus* | 0.275 | 1832.2 | - | - | - | - | - | - | - | - | - | - | - | - | - | X | - |
| *Erinaceus europaeus* | 0.145 | 771.0 | - | X | - | - | - | X | - | - | - | - | - | - | - | - | - |
| *Crocidura suaveolens* | 0.086 | 5.2 | - | X | - | - | - | - | - | - | X | - | - | - | - | - | - |
| *Crocidura leucodon* | 0.062 | 9.8 | - | X | - | - | - | - | - | - | X | - | - | - | - | - | - |
| *Suncus etruscus* | 0.242 | 2.1 | - | X | - | - | - | - | - | - | - | - | - | - | - | - | - |
| *Sorex minutus* | 0.036 | 4.5 | - | X | - | - | - | - | - | - | X | - | - | - | - | - | - |
| *Sorex araneus* | 0.195 | 10.0 | - | X | - | - | - | - | - | - | X | - | - | - | - | - | - |
| *Sorex samniticus* | 0.548 | 8.3 | - | X | - | - | - | - | - | - | X | - | - | - | - | - | - |
| *Neomys fodiens* | 0.818 | 14.3 | X | - | - | - | X | - | - | X | - | - | - | - | - | - | - |
| *Neomys anomalus* | 0.817 | 16.0 | X | - | - | - | X | - | - | X | - | - | - | - | - | - | - |
| *Talpa europaea* | 0.202 | 77.0 | - | X | - | - | - | - | - | - | - | - | - | - | - | - | - |
| *Rhinolophus ferrumequinum* | 0.130 | 21.1 | - | - | - | X | - | - | - | - | - | - | - | - | - | - | - |
| *Rhinolophus hipposideros* | 0.130 | 4.7 | - | - | - | X | - | - | - | - | - | - | - | - | - | - | - |
| *Tadarida teniotis* | 0.245 | 27.0 | - | - | - | X | - | - | - | - | - | - | - | - | - | - | - |
| *Eptesicus serotinus* | 0.616 | 22.9 | - | - | - | X | - | - | - | - | - | - | - | - | - | - | - |
| *Pipistrellus nathusii* | 0.132 | 7.3 | - | - | - | X | - | - | - | - | - | - | - | - | - | - | - |
| *Pipistrellus pipistrellus* | 0.938 | 5.7 | - | - | - | X | - | - | - | - | - | - | - | - | - | - | - |
| *Pipistrellus kuhlii* | 0.147 | 5.9 | - | - | - | X | - | - | - | - | - | - | - | - | - | - | - |
| *Pipistrellus pygmaeus* | 0.139 | 5.3 | - | - | - | X | - | - | - | - | - | - | - | - | - | - | - |
| *Nyctalus noctula* | 0.019 | 28.0 | - | - | - | X | - | - | - | - | - | - | - | - | - | - | - |
| *Nyctalus leisleri* | 0.027 | 13.3 | - | - | - | X | - | - | - | - | - | - | - | - | - | - | - |
| *Barbastella barbastellus* | 0.016 | 8.0 | - | - | - | X | - | - | - | - | - | - | - | - | - | - | - |
| *Plecotus austriacus* | 0.045 | 7.3 | - | - | X | - | - | - | - | - | - | - | - | - | - | - | - |
| *Plecotus auritus* | 0.023 | 7.8 | - | - | X | - | - | - | - | - | - | - | - | - | - | - | - |
| *Hypsugo savii* | 0.317 | 6.3 | - | - | - | X | - | - | - | - | - | - | - | - | - | - | - |
| *Vespertilio murinus* | 0.081 | 18.7 | - | - | - | X | - | - | - | - | - | - | - | - | - | - | - |
| *Myotis nattereri* | 0.006 | 7.1 | - | - | X | - | - | - | - | - | - | - | - | - | - | - | - |
| *Myotis mystacinus* | 0.006 | 5.0 | - | - | - | X | - | - | - | - | - | - | - | - | - | - | - |
| *Myotis myotis* | 0.079 | 24.8 | - | - | X | - | - | - | - | - | - | - | - | - | - | - | - |
| *Myotis blythii* | 0.079 | 22.5 | - | - | - | X | - | - | - | - | - | - | - | - | - | - | - |
| *Myotis bechsteinii* | 0.059 | 9.5 | - | - | X | - | - | - | - | - | - | - | - | - | - | - | - |
| *Myotis emarginatus* | 0.002 | 7.7 | - | - | X | - | - | - | - | - | - | - | - | - | - | - | - |
| *Myotis daubentonii* | 0.588 | 7.6 | - | - | - | X | - | - | - | - | - | - | - | - | - | - | - |
| *Miniopterus schreibersii* | 0.389 | 11.5 | - | - | - | X | - | - | - | - | - | - | - | - | - | - | - |
| *Vulpes vulpes* | 0.312 | 5476.2 | - | X | - | - | - | X | - | - | - | - | - | - | - | - | - |
| *Canis lupus* | 0.867 | 32183.3 | - | - | - | - | - | X | - | - | - | - | - | - | - | - | - |
| *Mustela putorius* | 0.355 | 915.4 | - | - | - | - | - | X | - | X | - | - | - | - | - | - | - |
| *Mustela nivalis* | 0.257 | 103.9 | - | - | - | - | - | X | - | - | - | - | - | - | - | - | - |
| *Martes foina* | 0.061 | 1540.8 | - | - | - | - | - | X | - | - | - | - | - | - | - | X | - |
| *Meles meles* | 0.407 | 13000.0 | - | X | - | - | - | X | - | - | - | - | - | - | - | X | - |
| *Lutra lutra* | 0.912 | 8785.1 | - | X | - | - | - | X | - | X | - | - | - | - | - | - | - |
| *Sus scrofa* | 0.003 | 96118.1 | - | - | - | - | - | - | - | - | - | - | - | - | - | X | - |
| *Capreolus capreolus* | 0.400 | 22500.0 | - | - | - | - | - | - | - | - | - | - | - | - | - | X | - |
| *Dama dama* | 0.343 | 52375.0 | - | - | - | - | - | - | - | - | - | - | - | - | - | X | - |


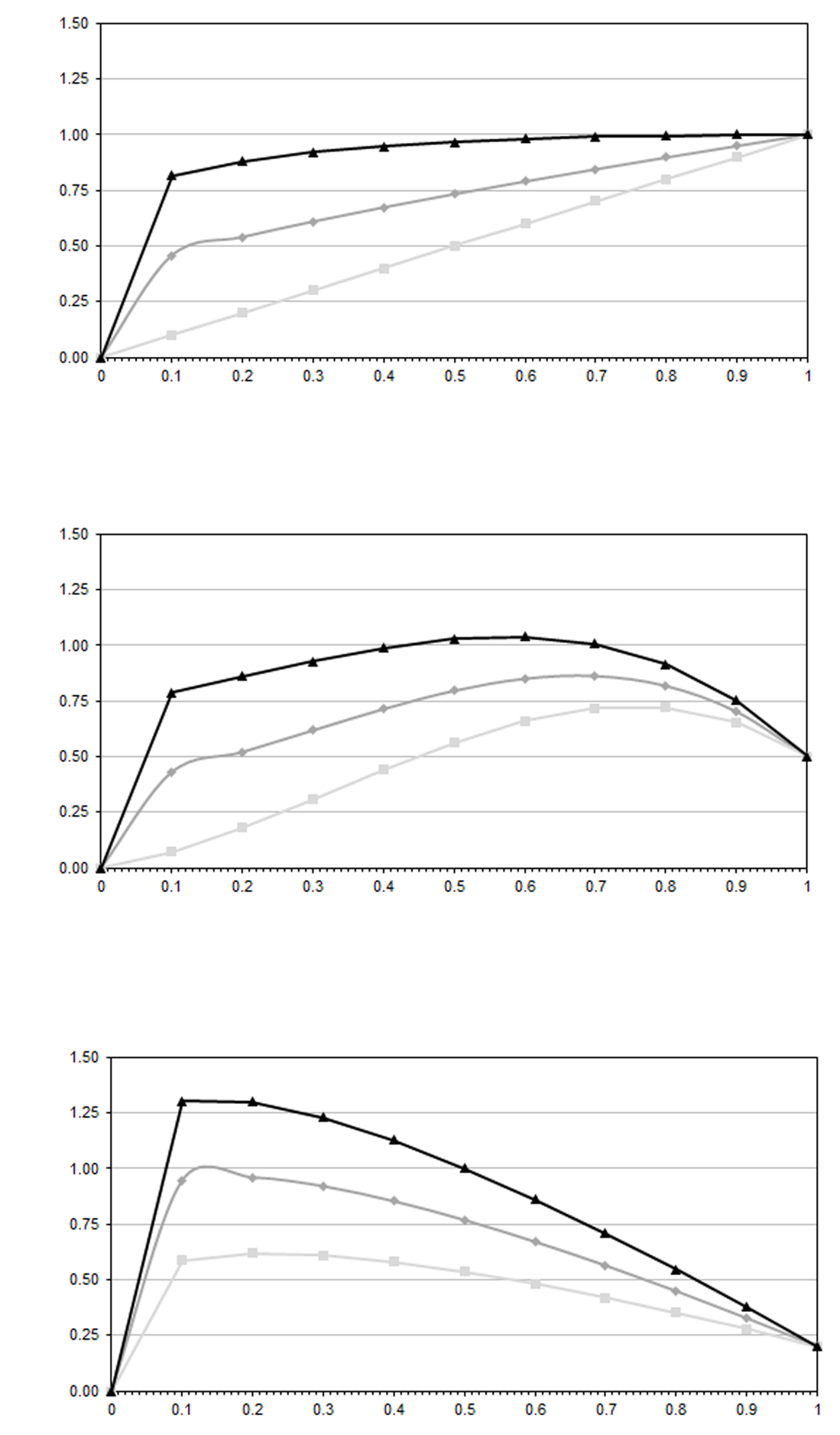


c)

b)

a)

Figure S1. Habitat suitability functions used in Expert-Based Models. Relative habitat suitability (y-axis) depends on the amount of a suitable (β_m_ = 1) main land-use class (x-axis) according to a) internal selection (Eq.1), b) internal edge selection (Eq.2a) and c) external edge selection (Eq.2b), when the suitability (β_s_) of the complementary land-use class is 0 (light grey curve), 0.5 (dark grey curve) or 1 (black curve).

**References**

Storchová, L; Hořák, D (2018), Data from: Life-history characteristics of European birds, Dataset, <https://doi.org/10.5061/dryad.n6k3n>
